# The Effect of Surface Treatment on Structural Properties of CVD Diamond Layers with Different Grain Sizes Studied by Raman Spectroscopy

**DOI:** 10.3390/ma14051301

**Published:** 2021-03-08

**Authors:** Anna Dychalska, Wojciech Koczorowski, Marek Trzcinski, Lidia Mosińska, Mirosław Szybowicz

**Affiliations:** 1 Faculty of Materials Engineering and Technical Physics, Poznan University of Technology, Piotrowo 3, 60-965 Poznan, Poland; wojciech.koczorowski@put.poznan.pl; 2 Centre for Advanced Technologies, Adam Mickiewicz University, ul. Umultowska 89C, 61-614 Poznan, Poland; 3 Institute of Mathematics and Physics, UTP University of Science and Technology, al. S. Kaliskiego 7, 85-796 Bydgoszcz, Poland; marekt@utp.edu.pl; 4 Institute of Physics, Kazimierz Wielki University, Powstańców Wielkopolskich 2, 85-090 Bydgoszcz, Poland; lidiamosinska@ukw.edu.pl

**Keywords:** diamond CVD, hydrogen treatment, Raman spectroscopy, XPS, SEM, CA

## Abstract

Extensive Raman spectroscopy studies combined with scanning electron microscopy (SEM) and X-ray photoelectron spectroscopy (XPS) measurements were performed to investigate structural and chemical changes in diamond layers deposited by chemical vapour deposition (CVD) upon post-growth treatment with hydrogen. The aim of this study is to characterize the changes in micro-structural properties of diamond layers with different grain sizes and different contents of sp^2^ carbon phase. Hydrogenation or oxidization of diamond layer surface is often performed to modify its properties; however, it can also strongly affect the surface structure. In this study, the impact of hydrogenation on the structure of diamond layer surface and its chemical composition is investigated. Owing to their polycrystalline nature, the structural properties of CVD diamond layers can strongly differ within the same layer. Therefore, in this project, in order to compare the results before and after hydrogen treatment, the diamond layers are subjected to Raman spectroscopy studies in the vicinity of a T-shape marker fabricated on the surface of each diamond layer studied.

## 1. Introduction

The usefulness of diamond layers due to their outstanding properties such as extreme hardness, electrochemical and biological stability is a well-known fact. Recently, much attention has been paid also to the surface properties of CVD diamond layers, because of the strong dependence of their electron affinity, surface conductivity and wettability on the type of terminating molecule [1,2].

In general, the two most common types of diamond surface terminations are those with oxygen or hydrogen species, in which the dangling bonds on the surface and in grain boundaries are terminated with these specific atoms. These O- and H- terminated diamond surfaces exhibit contrasting properties, i.e., the oxygen terminated surface shows high biological stability, high wettability, positive electron affinity (PEA) and no electric conductivity, while that terminated with hydrogen species shows the hydrophobic behaviour. Moreover, the H-terminated diamond surface exposed to air exhibits negative electron affinity (NEA) and p-type conductivity even without doping. This phenomenon was firstly observed by Landstrass and Revi [3]; it was later shown that the activation of the surface with NO_2_ leads to an enhancement of the hole conductive layer [4,5].

The possibility to produce diamond of different surface properties opens multiple potential applications for H- or O- terminated diamond surfaces. Interestingly, although their p-type conduction mechanism is still controversial, diamond layers have already been used to produce impressive FETs (field effect transistors) [6,7]. Hydrogen terminated diamond layers have also been used for diamond based cold cathodes [8,9]. On the other hand, oxygen terminated diamond layers, thanks to their ability to adsorb small molecules [10,11,12,13], have been used as electrodes for electrochemistry [14], or as sensing and delivery devices [15].

In comparison to other large band gap semiconductors, diamond surface is rather easy to functionalize and its properties are easy to modify [16,17]. The usual route to oxidise or hydrogenate a diamond surface is its exposure to oxygen or hydrogen plasma. To obtain a hydrogen-terminated surface, the activation of hydrogen atoms is performed usually by hot filament (HF) process [18,19,20], microwave (MW) process [21,22,23] or radio frequency (RF) process [22]. Recently, it has also been shown that contact electrification-induced hydrogenation method results in effective hydrogen termination of nanodiamond surface [24].

Additionally, it has been shown that also the exposure to molecular hydrogen fluxes at 800 °C can lead to hydrogen termination of (110) diamond surface [18], although this method was ineffective for the (100) diamond. Moreover, a study of single crystal diamonds with different orientations (100), (111), (110) exposed to MW deuterium plasma has shown that the surfaces of the highest and lowest concentrations of hydrogen were (110) and (100) one, respectively [25]. It has been shown that MW activated deuterium plasma can diffuse into the bulk diamond to the depth of 0.6 µm.

Variation of the diamond surface reactivity with different nature and orientation of diamond grains may be due to different surface area and different reconstruction of the surface [19]. One can see that the efficiency of the termination process depends on many factors, e.g. crystal orientation, the presence of crystallographic anisotropy.

In comparison to single crystal diamond, few papers have been published about influence of hydrogen treatment on polycrystalline diamond layers. One can expect that the diamond surface modification processes, irrespective of hydrogen or oxygen terminated surface, have impact both on the structural and surface properties of the diamond layer. Additionally, the extent of this impact should depends on the size of diamond grains or on sp^3^/sp^2^ hybridized carbon content.

In general, plasma treatment may lead to selective elimination of non-diamond carbons in a diamond layer as a result of different etching rates of sp^2^ and sp^3^ hybridized carbons. It has also been shown that hydrogen treatment can lead to 85–90% replacement of O by H but, at the same time, hydrogen-terminated surface is very stable [20].

The hitherto reports on the impact of hydrogen treatment on the diamond layer surface structure have been inconsistent. On the one hand, Shpilman et al. [26] have observed that the exposure of polycrystalline CVD diamonds to MW hydrogen plasma results in preferential etching of nondiamond carbon phases and formation of sp^3^-C–H - terminated surface. The authors of [23] have reported initial increase in the surface roughness with the time of plasma treatment but, for the longest time of hydrogen treatment application, they observed a decrease in this parameter. Additionally, the C-H concentration on the surface also increased, but all changes were very small—less than 1%. At the same time, XRD measurements before and after exposition of polycrystalline diamond layers to hydrogen plasma have not revealed any significant changes in their structure.

On the other hand, as reported in [22], the morphology of NCD film was not influenced by hydrogen treatment, but it was not the case for DLC film whose surface deterioration was observed. The efficiency of surface termination and its stability have also been reported to be higher for high temperature plasma.

As polycrystalline diamond layers can strongly vary in the size of diamond grains and quality over the sample, this inconsistency in the impact of hydrogen treatment on diamond surface may be due to, among others, different areas of a given investigated sample, as none of the authors of the above-mentioned reports compared results from the same area of diamond layers. Therefore, in this study, we aim to investigate exactly the same area of each sample to clarify the impact of hydrogen treatment on the surface and structural properties of diamond layers.

Wade et al. [27] have found that the electrical conductivity of H-terminated diamond surface increases with its roughness. However, it is probably not due to geometry of the diamond surface but it is rather a result of increased reactivity of the sites that generate carriers. Therefore, from the application point of view, it is crucial to determine the effect of the termination process on structural properties of diamond layers, in order to identify the properties affected by chemical and geometrical modifications from those induced by structural changes. It is worth noting that the change in the diamond structure observed after exposure to hydrogen plasma may possibly arise as a consequence of other process-related factors and not as a result of direct interaction between the surface and hydrogen atoms. For example, it has been observed that room temperature treatment with hydrogen resulted in getting highly defective structure, whereas the treatment with MW hydrogen plasma at ~1100 K, did not [28]. This RT treatment has been performed by capacitive coupled plasma with high bias, which could be the main reason for achieving higher defect density; however, low temperature or pressure of the process could have also played a role.

Raman spectroscopy is a very useful technique to investigate structural changes in carbon materials, because it is highly sensitive to the type of carbon bonds hybridization—sp^2^ and sp^3^ phase, both in amorphous and crystalline phase. Additional information can be also extracted from the photoluminescence PL background of the Raman spectrum, as it has been shown that the slope *m* of the linearly subtracted PL background normalized to the intensity of G band—*m/I_G_* is proportional to the hydrogen concentration in hydrogenated amorphous carbon [29]. It has also been shown that the PL background of Raman spectra of diamond layers depends on the hydrogen concentration at grain boundaries and on the surface [30,31]. However, density of defects in the diamond layer also influences the PL background behaviour and possible changes in *m/I_G_* parameter might be very small; therefore, data interpretation must be performed with caution.

Changes in the Raman spectra after hydrogenation process have been rarely reported, most likely due to their subtlety and problems with their detection if measurements are taken in different spots of the sample. For example, as reported in [22], the G band in the Raman spectrum of a diamond layer after hydrogen treatment has been slightly moved to lower wavenumbers, which might indicate an increase in the fraction of the sp^3^ carbon phase, possibly in C-H bonds. Therefore, in our study, subsequent Raman measurements after each surface treatment were always performed in the same area of the sample. This approach should help in disclosing contributions coming from chemical or structural changes, which should guarantee high data reliability, even if observed changes are relatively small.

In this study we consider four diamond layers with different average grain sizes, subjected to hydrogen termination as the first scheduled treatment, (the next planned treatment is oxidation) and we analyse the changes induced by this process in the same micro areas of the samples. Identification of the same area for each sample after any process was possible thanks to fabrication of a characteristic T-shaped marker on each diamond layer surface.

The aim of this work is to investigate the impact of hydrogen treatment on structural and chemical properties of CVD diamond layers with different grain sizes through analysis of evolution of diamond, D, G bands and photoluminescence background of Raman spectra. Additionally, the sample surface morphology was analysed by the SEM technique, while the chemical compositions of the layers were established by the XPS technique and contact angle measurements.

## 2. Experimental

For this study, four diamond layers were deposited on silicon (100) substrates by HF CVD process with dimensions of 5 × 5 mm^2^ and thickness of 0.5 mm. To ensure effective nucleation of diamond precursors, priori deposition process silicon substrates were mechanically scratched by diamond powder having grain sizes of 0.2 μm and then cleaned in methanol. The working gas consisted of hydrogen gas H_2_ (at least 96%) and methane CH_4_. For deposition process tungsten filament has been used. In order to achieve different grain sizes of diamond crystallites, parameters such as the time of deposition and concentration of methane and hydrogen in working gas varied, as shown in Table 1. For all samples during deposition process the pressure of working gas was kept at 27 mbar, whereas the temperatures of the substrate and filament were set to 900 K and 2000 K, respectively. Additionally, in Table 1 for all samples the estimated thickness of diamond layers is presented (based on the growth rate of the deposition process).

**Figure 1:**
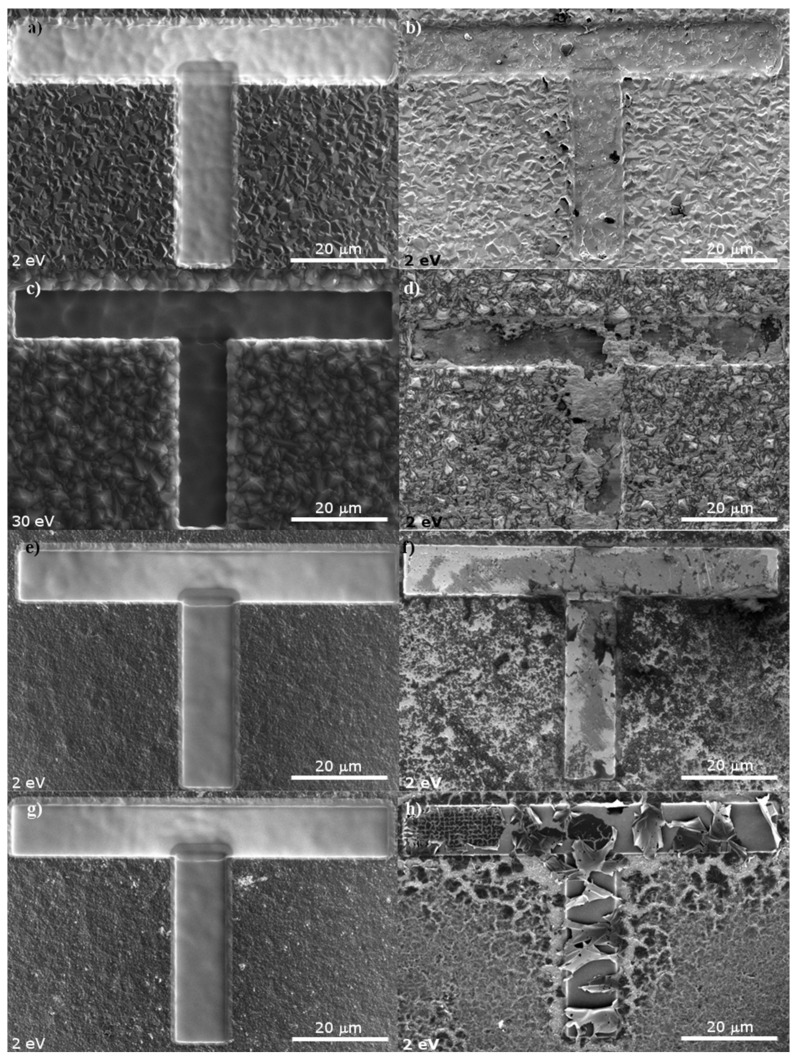
SEM micrographs of diamond layer surface before (left panel) and after hydrogen treatment (right panel) for samples (**a**–**b**) MCD2, (**c**–**d**) MCD2.5, (**e**–**f**) NCD0.2, (**g**–**h**) NCD0.02.

After the deposition process, the initial Raman, SEM and XPS measurements for the diamond layers were performed. The next step was to mark specific areas on the diamond layers to ensure that further measurements will be always performed in the same location on the sample. The T-shape markers on the sample surface were made using the Focussed Ion Beam—FIB patterning technique using Ga+ ion with the energy 30 keV and ion current in the range of 9–21 nA (FEI Helios NanoLab 660, FEI, Eindhoven, North Brabant, The Netherlands). The results are presented in Figure 1.

The post-growth process of hydrogen treatment was carried out in a CVD chamber by exposure of diamond layers to pure hydrogen with working filament, which ensured activation of H_2_ to radical atoms. The hydrogen treatments were performed for 15 min from reaching a suitable pressure (30 mbar); during this process, the filament temperature was kept at 2300 K and that of the diamond layers at 1100 K.

**Figure 2:**
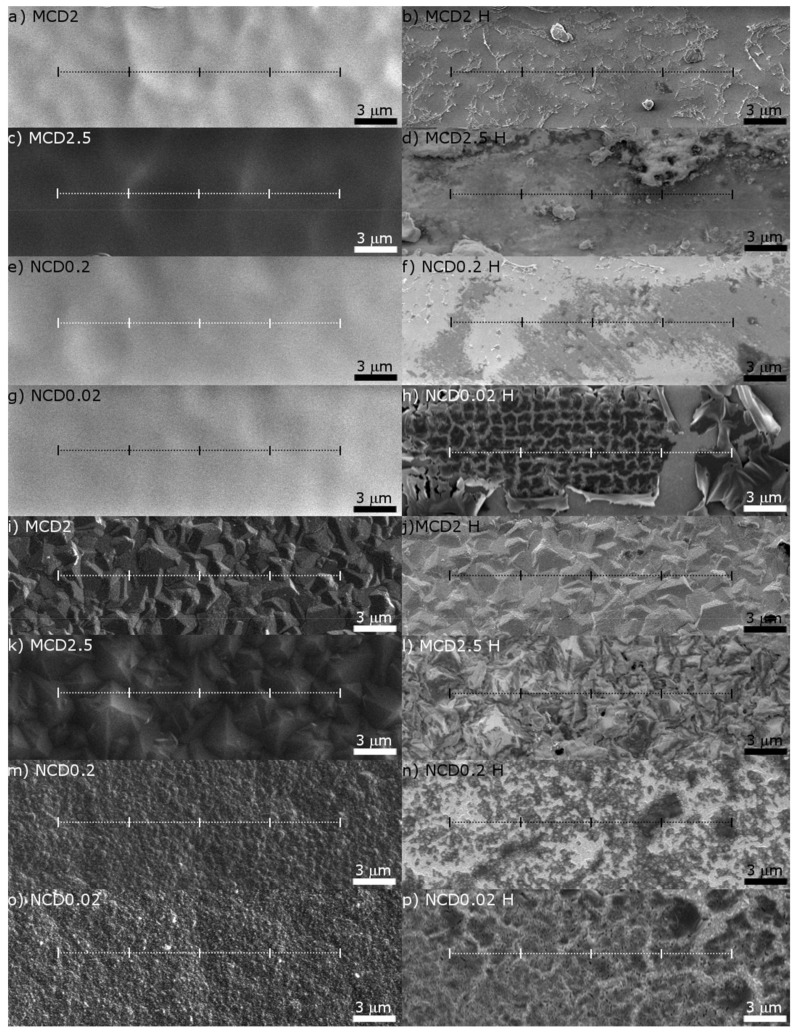
The position of linear Raman map (**a**–**h**) in the marker, and (**i**–**p**) outside the marker. The as-deposited diamond layers are shown on the left side of the figure, while the layers after hydrogen treatment are given on the right side. The line length is 20 µm, short vertical lines indicate the points at which the Raman spectra were taken.

Raman measurements were performed before and after hydrogen treatment in the same area with 488 nm excitation light, by Renishaw in via Raman system with a conjugated confocal microscope (Leica Microsystems, Wetzlar, Germany). For each sample, two linear maps were collected with a 5 µm step (from the inside and outside of the marker, as shown in Figure 2), and each map consisted of five spectra.

The spectra were recorded in a wide range, i.e., from 100 to 8000 cm^−1^, to also enable the analysis of photoluminescence behaviour. In the used micro-Raman system, the configuration for blue laser light yields a spatial resolution of about 1 µm. In this paper, we used pseudo-Voigt curves for all bands considered, to provide the best fit of experimental points and to easily determine the PL background slope. Additionally, such a fitting procedure allows bands to naturally change their parameters as a result of structural changes [32].

The XPS measurements were performed in the UHV chamber with a base pressure ≤ 2 × 10^−10^ mbar. Monochromatic Al Kα X-ray source (ħω = 1486.6 eV) was used to excite the photoelectrons from the surface area of the samples. The energy of photoelectrons with energy resolution ΔE = 100 meV was measured by VG-Scienta R-3000 hemispherical analyser (Scienta Omicron, Uppsala, Sweden).

Scanning Electron Microscope (SEM) images were recorded by a FEI Helios NanoLab 660 tool (Gaithersburg, MD, USA). The SEM images were collected at electron beam energy up to 30 kV (usually at 2 kV).

## 3. Results and Discussion

Figure 1 presents micrographs of a diamond layer surface before (left side) and after (right side) hydrogenation. As shown on the left side of Figure 1, the fabrication of a T marker did not impact the area beyond the marker. However, in the T shaped area, amorphous-like structures can be observed, regardless of the diamond grain sizes in the layer.

After hydrogen treatment, the topological structure of the diamond layer surface changes, as shown in the right side of Figure 2 (magnification of Figure 1); for MCD2 and MCD 2.5 the amorphous phase from within the marker is almost entirely etched away. In some sites of MCD2 sample (Figure 2b) also chain-like structures are clearly visible, whereas in MCD 2.5 sample (Figure 2d) the material remaining after the removal of the amorphous carbon is in the form of agglomerates. Interestingly, in nanocrystalline samples (Figure 2f,h) the treatment with hydrogen caused delamination of the amorphous carbon/nanodiamond layer within the marker.

Diamond crystallites in samples MCD2 and MCD2.5 do not change significantly after hydrogenation; however, it seems that for the latter sample, an additional layer of amorphous carbon covers the diamond grains and some slight modification and partial removal of smaller structures in grain boundaries have taken place (Figure 2k,l). Similar coverage can be seen for NCD0.2 sample.

In Figure 2i,k the difference in the geometry of diamond grains between sample MCD2 and MCD2.5 is also visible, which would imply different preferential orientation of these samples surfaces. Although the diamond grain sizes of samples MCD2 and MCD2.5 are similar, the degree of modification of the latter sample was higher, which suggests that the effect of the preferential orientation of the surface was greater than that of the grain size.

For nanocrystalline samples significant changes in the structures outside the maker are also manifested as cracks on the surface and even delamination of carbon layer in NCD0.02 (Figure 2m,p). It seems that for diamond layers of smaller grain size and higher sp^2^ concentration, the impact of hydrogen treatment on the surface structure is greater. Moreover, in the case of sample NCD0.2, after hydrogenation, the area within the marker (Figure 2f), looks to be more uniform, as regards surface morphology in comparison to the microcrystalline samples. This suggests that for this nanocrystalline sample, all post-ion etched amorphous layer was removed, which was not the case for microcrystalline samples. On the other hand, for sample NCD0.02 graphitization and delamination of post-ion etched amorphous layer is observed upon hydrogenation in Figure 2h).

**Figure 3:**
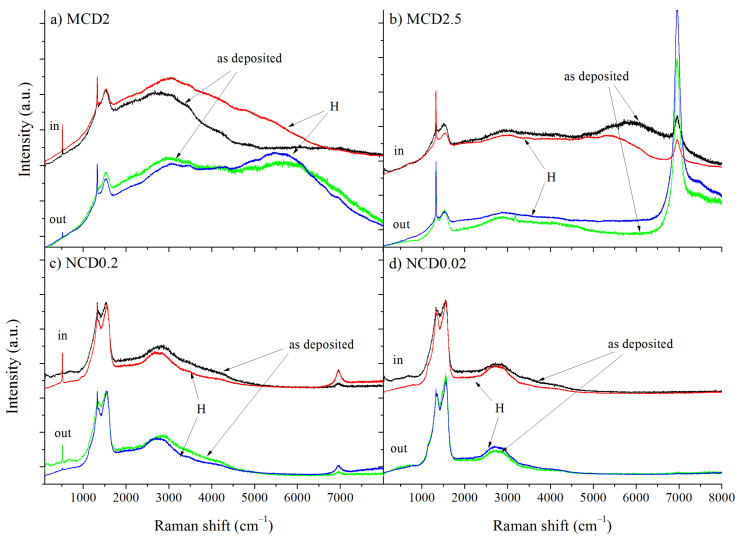
Raman spectra of diamond layers (**a**) MCD2, (**b**) MCD2.5, (**c**) NCD0.2, (**d**) NCD0.02. Black and green line—before hydrogen treatment, red and blue line—after hydrogen treatment.

**Figure 4:**
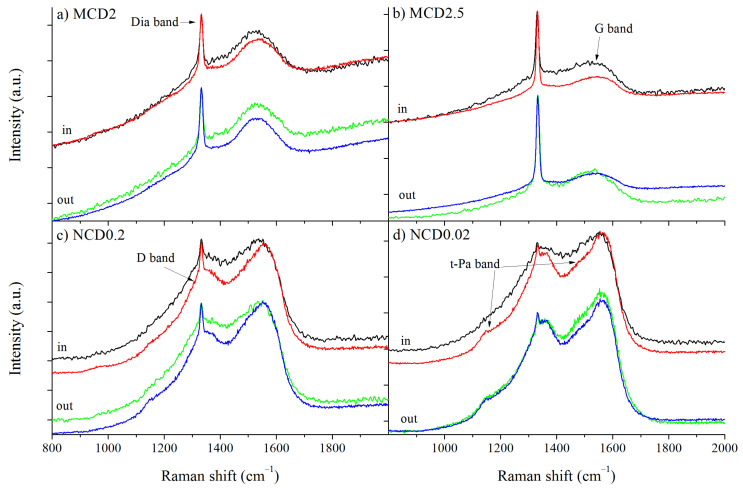
The first order Raman spectra of diamond layers with indication of diamond, D, G, t-Pa band positions: (**a**) MCD2, (**b**) MCD2.5, (**c**) NCD0.2, (**d**) NCD0.02. Black and green lines- before hydrogen treatment, red and blue line—after hydrogen treatment.

The Raman linear maps were made for two sites: inside and outside the marker, as indicated in Figure 2. Additional spectra were taken at random locations of samples to determine whether or not the fabrication of the marker had influenced the overall structure of the diamond layers. No significant differences were observed between the Raman spectra taken at random locations of a given sample and the spectra from the linear map outside the marker. The Raman spectra taken at the same point of each diamond layer shown in Figure 3 and Figure 4 (all spectra are normalized to diamond band) imply that the fraction of the non-diamond phase is reduced after exposition to hydrogen, which is evidenced by the decrease in the signal intensity in the region 1100–1600 cm^−1^, where non-diamond related bands appear (i.e., t-Pa, D an G bands). The presence of amorphous-like structures inside the T-shaped area before hydrogenation is also evidenced by Raman measurements, manifested by an increased a noise level in the spectrum.

Only for sample MCD2.5, very strong luminescence from the Si vacancy-related defect (SiV) appears at around 7000 cm^−1^ as shown in Figure 3b. This exception probably can be caused by higher concentration of Si impurities from silicon substrate in the chamber during deposition process of this sample, and/or by preferential incorporation of silicon in MCD2.5 due to the orientation of diamond grains.

Additionally, the photoluminescence background exhibits some changes after hydrogen treatment. Interestingly, the Si vacancy-related defect for MCD2.5 sample is more prominent outside the marker. This could be a result of efficient ion etching of Si impurities within the marker at the stage of the marker formation, leading to partial elimination of defects. In nanocrystalline diamond layer, the changes in the shape of PL background are smaller. The spectra of all microcrystalline samples (MCD) exhibit changes in PL background intensity after hydrogenation irrespective of whether the measurements were taken inside or outside the marker. Additionally, in the Raman spectrum taken outside the marker for MCD2 a wide PL band centred at 5700 cm^−1^ (~1.8 eV) is present (also seen for as-deposited MCD2.5 in the marker), which becomes more prominent after hydrogenation. Such a broad PL band with a maximum emission at 1.8–2.2 eV is characteristic for amorphous carbon materials and it is most likely related to recombination processes within sp^2^—hybridized carbon clusters in sp^3^ matrix [33]. A similar band has also been observed for diamond layers with sp^2^ admixture [34]. As the position and the width of this PL band can differ due to changes in amorphous carbon structure, the treatment with hydrogen most likely induced locally structural changes in sp^2^ admixture. In contrast, the spectra taken for the nanocrystalline samples NCD0.2 and NCD0.02 do not exhibit strong photoluminescence features, which may be connected to the lower concentration of hydrogen in the amorphous carbon phase. Another explanation is that the NCD layers tend to be more defective due to higher volume of grain boundaries; thus, the less intense PL background may also indicate higher defect concentration, which leads to non-radiative recombination.

Figure 4 presents the first order Raman spectra of the diamond layers before and after hydrogen treatment. A sharp line observed at around 1332.5 cm^−1^ is assigned to triply degenerated phonon of diamond lattice at the centre of Brillouin zone [35]. As the CVD diamond layer can be considered as a mixture of both diamond crystals and amorphous carbon, other bands are also observed. The D bands appear at around 1340–1390 cm^−1^ and G band at around 1510–1590 cm^−1^, the first arise due to breathing mode of carbon rings and is also related to double resonance effect, whereas the second band is assigned to the stretching mode of carbon atoms both in rings and chains [36].

Therefore, the intensity ratio of D and G bands: *I_D_/I_G_* (not integrate intensity) indicates the degree of structural ordering, whereas the G band position may indicate the fraction of sp^3^ carbon phase [36,37]. In the spectra of all samples D and G bands are evidently present; however, for the nanodiamond layers they are much more prominent because of a higher content of amorphous carbon phase.

In the Raman spectra of diamond layers, the bands related to trans-polyacetylene like structures are often observed [38,39,40,41,42], appearing at 1150 cm^−1^ (t-Pa1 band) and at 1485 cm^−1^ (t-Pa2 band). These bands are associated with the bending mode of C-H in sp^2^ hybridization carbon and the stretching mode of the C=C in a linear chain, respectively. Again, these bands are clearly observable for nanocrystalline samples. For the sake of consistency, these bands in the spectra of microcrystalline samples were also included in the fitting procedure.

In this study, the slope *m* of the PL background in the Raman spectrum was linearly subtracted in the range from 900 to 1800 cm^−1^. The slope *m* normalized to the intensity of G band i.e., *m/I_G_* parameter (in μm units) is the measure of radiative recombination of electron hole pairs within sp^2^ bonded clusters that occurs upon exposition to laser light. Hydrogen is believed to saturate non-radiative recombination sites like dangling bonds and, as a result, form centres of radiative recombination. Therefore, *m/I_G_* parameter may indicate the concentration of hydrogen in diamond layer [29,30,31].

On the other hand, the quality of diamond layer can be easily assessed by Quality Factor Q(A), which is approximately the percentage of sp^3^ hybridized carbon in the form of crystallites. The Quality Factor is calculated as the ratio between integrated intensity of diamond band in Raman spectra, to the sum of integrated intestines of all bands in Raman spectra, with the correction for different cross section of Raman scattering process for sp^2^ carbon [43]:

(1) $$Q\left(A\right)=\frac{A_{dia}}{A_{dia}+\frac{A_{sp^{2}}}{75}}\times 100\%$$

where $A_{dia}$ is integrated intensity of diamond band and $A_{sp^{2}}$ is the sum of integrated intensities of all sp^2^ related bands in the Raman spectra.

**Figure 5:**
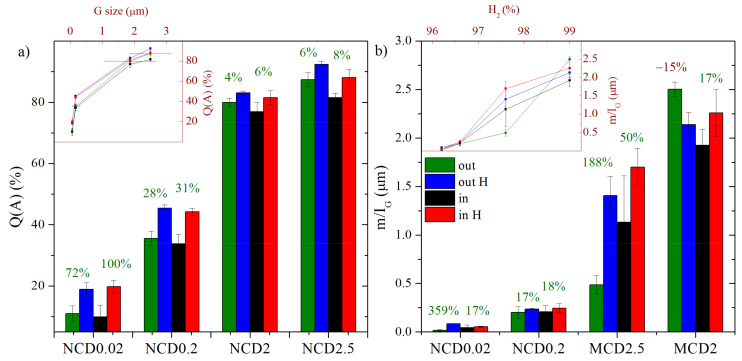
Changes in (**a**) quality factor Q(A) and (**b**) *m/I_G_* parameter, upon hydrogen treatment of the diamond layer. Green and blue stacks—average values for measurements taken outside the marker, before and after hydrogenation, respectively. Black and red stacks—average values for measurements taken in the marker, before and after hydrogenation, respectively. The insert plot in (**a**) presents the dependence of Q(A) on grain size of diamond layers and in (**b**) the dependence of *m/I_G_* on hydrogen concentration in the processing gases. Green or red numbers in plots (**a**) and (**b**) indicate an increase or decrease in Q(A) and *m/I_G_* values after hydrogenation.

The calculated Q(A) of diamond layer for samples MCD2.5, MCD2, NCD0.2 and NCD0.02 are as follows: 90%, 80%, 40% and 20%. Figure 5 presents the mean values of quality factor Q(A) and *m/I_G_* parameter for each sample before and after hydrogen treatment, both inside and outside the marker.

From the insert plots in Figure 5a,b, it becomes evident that with decreasing hydrogen concentration in the processing gas, the *m/I_G_* parameter decreases and the quality of the diamond layers deteriorates. The quality of diamond layer, expressed as approximated concentration of diamond crystalline forms in a sample, depends mainly on the size of diamond grains. It is well known that with decreasing grain size, the overall volume of grain boundaries increases and therefore the volume of the non-diamond phase in the sample (non-diamond concentration is 100%-Q(A)) increases. For all samples, regardless of hydrogen treatment, the content of amorphous phase is reduced leading to a higher quality of the layer (Figure 5a). For nanocrystalline samples, the increase in Q(A) is two-fold and, for other samples, it is less prominent (by 6–8%), since the Q(A) has been fairly high from the start. In general, for all samples inside the marker the Q(A) factor was slightly smaller, due to an additional amorphized carbon layer appearing after ion etching, and therefore, improvement of Q(A) factor was more noticeable.

Post-growth hydrogen treatment clearly leads to an increase in *m/I_G_* parameter (Figure 5b), which is understandable, given the fact that near the surface the layer was probably enriched in hydrogen; moreover, high temperature could result in reduction of defects. The only exception is sample MCD2 for which a decrease in *m/I_G_* parameter outside the marker was observed (by ~15%), which could be a result of the already mentioned defects generation in this area. In general, sample MCD2.5 exhibits one of the greatest improvement in *m/I_G_* parameter; also, NCD0.02 outside the marker shows an increase of 360%. For the other samples, the increase was lower and took a similar value of about 17%.

**Figure 6:**
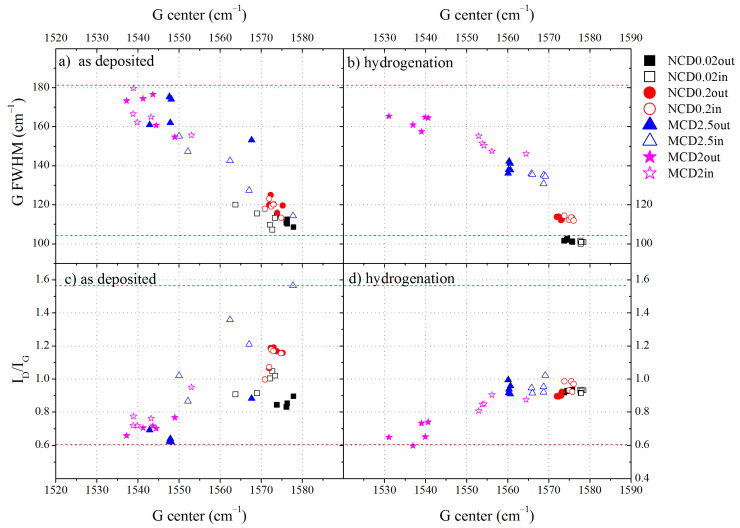
The dependence of (**a**–**b**) G FWHM and (**c**–**d**) *I_D_/I_G_* ratio on the G band position for all measurements points. Data collected before hydrogen treatment (**a**) and (**c**) are shown in the left panel, while those collected after hydrogen treatment (**b**) and (**d**)—in the right panel. The filled symbols represent the results obtained outside the marker, empty symbols—those obtained inside the marker.

Physical and chemical properties of amorphous carbon materials strongly depend on the fraction of the sp^3^ phase, hydrogen bonds concentration and on different possible clustering and orientation of the sp^2^ carbon phase. As micro- and nano-diamond layers often contain large amounts of amorphous carbon, which modifies their properties, the configuration of amorphous carbon also contributes to the final properties of the diamond layer. Therefore, in order to characterize the structure of non-diamond phase in diamond layers, the relations of the G band position, its FWHM and the *I_D_/I_G_* ratio were analysed for the samples before (Figure 6a,c) and after (Figure 6b,d) hydrogenation (empty icons—spectra taken at a site inside the marker, filled icons—outside it).

On the basis of Figure 6a, we can conclude that the amorphous carbon structure varies with deposition parameters. The wider the FWHM and the lower the position of G band, the greater the disordering of the amorphous carbon and, therefore, the higher the contribution of the sp^3^ amorphous carbon phase. We can assume that the level of ordering of the amorphous carbon phase increases with growing CH_4_ concentration, which would imply smaller content of the sp^3^ amorphous carbon phase and hydrogen; a similar finding has been reported in [44]. This observation is in good agreement with the estimated *m/I_G_* parameter, which also becomes smaller with increasing CH_4_ concentration.

The spectra taken inside the marker indicate that amorphous carbon there tends to be slightly more ordered than outside the marker, both before and after hydrogenation. In general, we observe that after hydrogen treatment, the amorphous carbon in diamond layers becomes even more ordered, however for MCD2 sample outside the marker, the reverse trend is observed, the amorphous carbon becomes even more disordered. Probably, hydrogenation induces the ordering of amorphous carbon due to thermal effects. During the termination process, the diamond layers reached a temperature of 1100 K, which could lead to ordering, as the formation of graphite crystallites due to high temperature annealing was observed in diamond layers [45] even in H_2_/Ar [46]. On the other hand, hydrogen termination should rather lead to more disordered structures. Most likely, two interplaying effects take place: the ordering of amorphous phase induced by high temperature and generation of sp^2^ or sp^3^ C-H bonds.

In the Raman spectroscopy with visible light excitation, the sp^3^ phase of amorphous carbon is not directly probed. However, from the three-stage trajectory of amorphization [36] we can deduce that the non-diamond phase in our diamond layers is mainly in the form of nanocrystalline graphite and amorphous carbon (stage 2). According to this model, the *I_D_/I_G_* ratio in stage 2 is inversely proportional to the fraction of the sp^3^ amorphous carbon phase. As follows from the analysis of FWHM, position of G band and *I_D_/I_G_* ratio, sample MCD2 contains amorphous carbon of which more than 10% is the sp^3^ hybridized carbon. The contribution of the amorphous sp^3^ phase continuously decreases with increasing CH_4_% in the working gas and for sample MCD2.5 (outside the marker) it is approximately 10%. Samples NCD0.2 and NCD0.02 contain less than 5% of the sp^3^ carbon phase. The surface inside the marker tends to show a lower content of the sp^3^ phase. After hydrogenation, the structure ordering increased and the content of the sp^3^ phase decreased to less than 5% for all samples, but MCD2.

**Figure 7:**
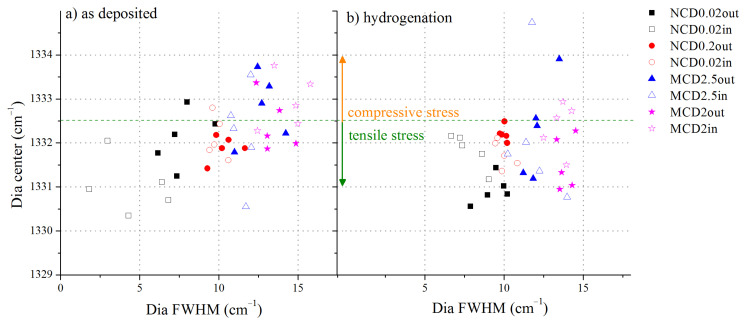
The relation between the diamond band position and its FWHM for all measurements points, (**a**) before hydrogen treatment, (**b**) after hydrogen treatment. Filled symbols represent results of measurements taken outside the marker, empty points—inside the marker.

To further investigate the quality of diamond layers, the diamond band position versus the diamond band FWHM was analysed, see Figure 7.

Before hydrogenation, microcrystalline samples tend to exhibit stress of compressive character, whereas nanocrystalline samples exhibit tensile stress. Although nanocrystalline samples have higher volume of grain boundaries, the FWHM of the line assigned to diamond in the Raman spectra of these samples is smaller than that of the analogous line recorded for microcrystalline samples. This would imply that the level of stress in the former samples is lower. However, it is highly unlikely that in the diamond layer with a high content of non-diamond phase, the FWHM of the diamond band would be as low as 2.5 cm^−1^ (sample NCD0.02). Thus, for the nanocrystalline sample NCD0.02 the reason for so small FWHM may be a splitting of the diamond band and obstruction of one sub-band by another component of the spectrum, e.g., a D band. Another reason may be the fact that microcrystalline layers are less uniform in size of diamond crystals. Indeed, the smallest structures in MCD2 and MCD2.5 are also in nanometre scale, and thus the higher value of FWHM would indicate more variation of diamond crystals sizes in a sample. After hydrogenation, the average FWHM value increases, but at the same time the stress becomes more of tensile character for all samples. This observation is consistent with the findings that hydrogen can lead to tensile stress [47] or to relaxation of stress by termination of dangling bonds in the sp^3^ amorphous phase [48].

**Figure 8:**
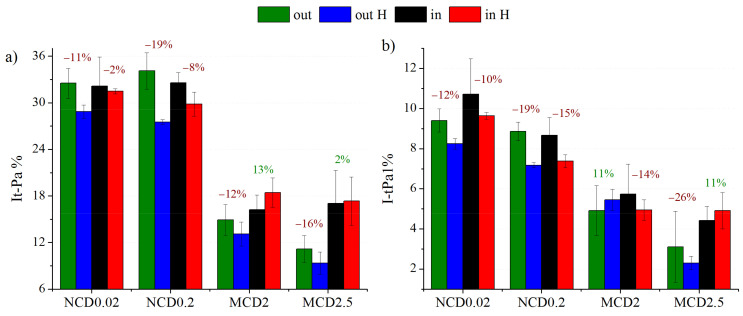
The percentages of (**a**) t-Pa and (**b**) t-Pa1 (at 1150 cm^−1^) bands in the Raman spectra. Green and blue stacks—average values for measurements taken outside the marker, before and after hydrogenation, respectively. Black and red stacks – average values for measurements taken within the marker, before and after hydrogenation, respectively. Green or red numbers in plots (**a**) and (**b**) indicate an increase or decrease in t-Pa or t-Pa1 contribution in Raman spectra after hydrogenation.

In this work, we also studied the impact of hydrogen treatment on trans-Polyacetylene structures. As follows from Figure 8a, hydrogenation results in reduction of the contribution of t-PA bands in the Raman spectra (calculated as the ratio of the sum of t-Pa1 and t-Pa2 bands intensities to the sum of intensities of all diamond layer spectra components).

For nanocrystalline diamond layers, a significant decrease upon H treatment in the contribution of trans-Polyacetylene structures is clear. Usually, this decrease is more pronounced for the samples with initially higher concentration of these structures. When measurements were made within the marker, the changes in the t-Pa related bands were not that obvious, and for MCD2 even an increase the t-Pa band contribution to the Raman spectrum was observed. This exception is interesting as far as the chain-like structures in sample MCD2 are concerned (Figure 2b). Of course, t-Pa structures are too small to be observable by SEM; however, the existence of bigger structures may imply favourable conditions for formation of chain-like structures. The band t-Pa1 at approximately 1150 cm^−1^ is often associated with C-H bonds in trans-Polyacetylene chains (sp^2^ hybridization). For the nanocrystalline diamond layers we observe also a reduction in the contribution of this band (Figure 8a). In the process of hydrogenation the chains are most likely broken and form short structures with sp^3^ carbon. The decrease in the number of C-H bonds in sp^2^ hybridization does not have to automatically mean that the number of C-H bonds in sp^3^ hybridization also decreases.

Slightly different behaviour is observed for MCD2.5 within the marker and for MCD2 outside the marker; however, the differences are in the range of error and are, therefore, negligible. It also seems that the area within the marker is more resistant to t-Pa removal then the outside area, probably because it is already highly modified.

**Figure 9:**
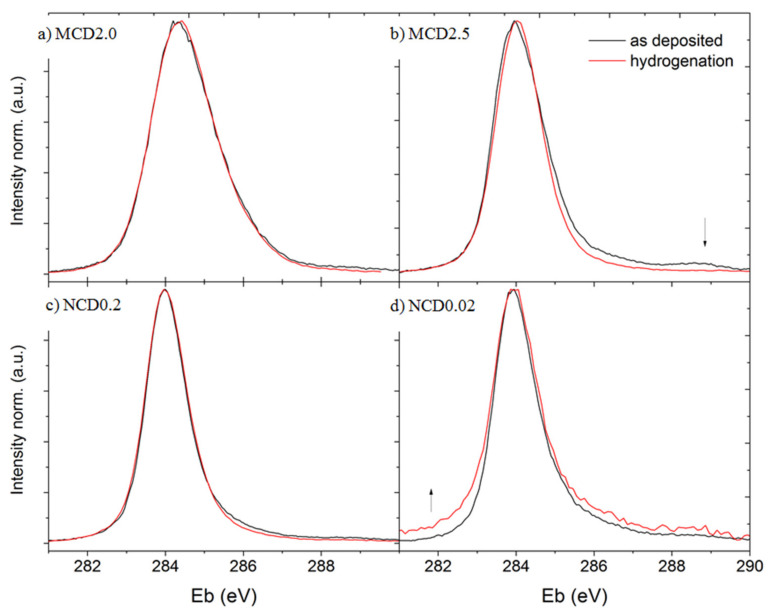
C1s peaks from XPS spectra of diamond layers before and after treatment exposure to hydrogen (black and red line, respectively) (**a**) MCD2, (**b**) MCD2.5, (**c**) NCD0.2, (**d**) NCD0.02.

To complete the analysis of hydrogen treatment effects, let us consider now chemical composition of diamond layer surface, as well as this surface wettability before and after hydrogenation. Figure 9 presents the XPS spectra of diamond layers, black line represents the data collected before hydrogenation, and red—after this treatment.

The width of C1s peak roughly corresponds to the amount of disordered carbon and is in good agreement with the Raman data, indicating that sample MCD2 is the one with the most disordered amorphous carbon phase. For MCD2.5 and NCD0.2, the C1s peak intensity decreases in the spectral range in which the signals assigned to oxygen carbon groups occur. Only for NCD0.02 sample an increase in the C1s peak intensity in this range is observed, which most likely implies a partial failure of the hydrogenation process, or rapid oxidation following this treatment. The presence of oxidized carbon bands in the C1s peak of diamond layers is expected as the diamond layer surface always contains oxygen-carbon groups when exposed to air.

**Figure 10:**
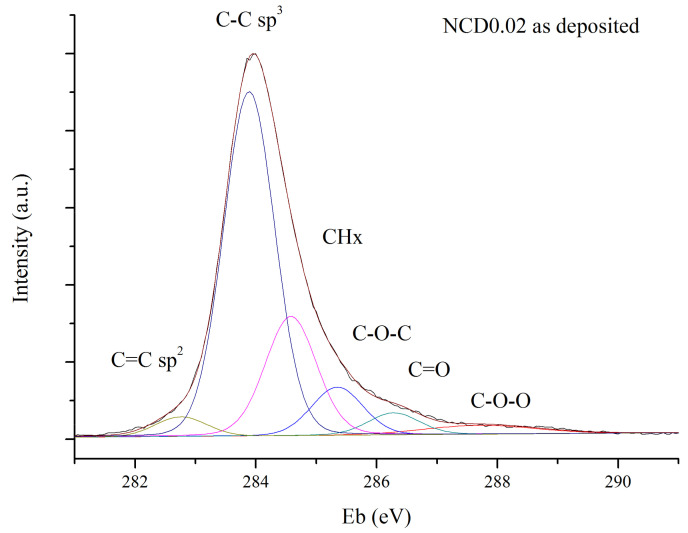
Exemplary C1s XPS spectra deconvolution for diamond layers NCD0.02, before hydrogenation.

Figure 10 shows an example of deconvolution of the C1s peak. The subtraction of linear background was applied to ensure the reproducibility of XPS spectra fitting. For all curves, the FWHM was set to 1 eV except for the last one, to cover up potential contribution from higher oxidation states of carbon or from π satellites.

The same initial parameters of fitting curves were used in deconvolution process of the spectra obtained before and after annealing, to ensure that the obtained results would be comparable, irrespective of the physical meaning of the curves.

In the XPS spectra of diamond, C1s peak appears at about 285.5 eV from that assigned to the bulk sp^3^ hybridized carbon; however, the position of C1s peak also strongly depends on charging effects and band bending. Therefore, in this study, we consider the relative positions of the bands, rather than the exact position of the C1s peak. The band shifted from the C–C sp^3^ signal by 0.75–0.95 eV towards lower binding energy is attributed to the sp^2^ hybridized carbon. The band at a higher binding energy, i.e., at 0.5–0.7 eV from that assigned to the sp^3^ C–C, might be associated with CH_2_ or CH_3_ bonds [49].

Usually, in the XPS spectra of carbon materials the CHx band is not fitted as it is presumably small, and close to the much bigger sp^3^ C–C peak [17,50,51]. However, diamond films deposited by CVD technique are inherently terminated by hydrogen, because during deposition process hydrogen makes 92–99% of all reactive gases [52]. Hydrogen atoms bond with amorphous carbon phase as well terminate the growing surface and prevent transformation of diamond structure into graphite-like structures [53]. Therefore, it is reasonable to assume that CHx component should be present in C1s peak, even for as-deposited diamond layers, as noted by other authors [23,54].

The bands shifted towards higher energies from that corresponding to C–C sp^3^ by 1.3–1.5 eV and by 2.3–2.5 eV originate most likely from C–O–C or –C–OH and –C=O or O–C–O groups, respectively [51]. Additional peak, observed at the highest energies, may be attributed to C–O–O, or to π satellites.

The only restriction of the fitting procedure was the fact of relative energy differences between bands, as mention above. When six curves were taken into account, a very good fit to the experimental data was obtained, as one can see in Figure 10.

**Figure 11:**
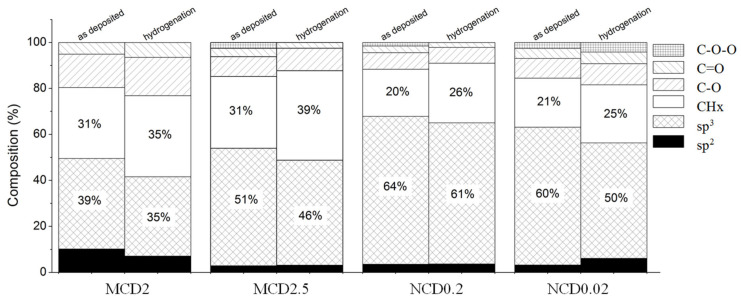
The relative contribution of C–C sp^2^, C–C sp^3^ CHx, C–O–C, -C=O and C–O–O components as derived from the peak fitting procedure for diamond layers MCD2, MCD2.5, NCD0.2, NCD0.02 before and after hydrogen treatment.

The relative contributions of different carbon species to the C1s peak before and after hydrogenation are shown in Figure 11 and also listed in Table 2.

From among the as-deposited diamond layers, sample MCD2 has the smallest concentration of sp^3^ carbon from amongst all samples, despite the fact that it has the second largest diamond grains. At the same time for this sample the highest concentration of sp^2^ carbon has been observed. This indicates that diamond grains in sample MCD2 may be covered by relatively thick layer of partially hydrogenated amorphous carbon, which decreases the signal from sp^3^ hybridized carbon.

Microcrystalline samples have higher concentration of CH_2_ or CH_3_ bonds on the surface, which is consisted with Raman results. For all samples after hydrogenation, the increase in the presumably CHx related band contribution in C1s peak is observed, at the expense of that of the sp^3^ phase. This result implies that new hydrogen bonds are formed mainly due to the etching of nanocrystalline phase of diamond, and not of amorphous phase at the grain boundaries. Such occurrence might be due to both, complete removal of amorphous carbon phase by hydrogen, or ordering of amorphous carbon phase, i.e., transformation of sp^3^ to sp^2^ configuration, which is also observed in the Raman spectra.

**Figure 12:**
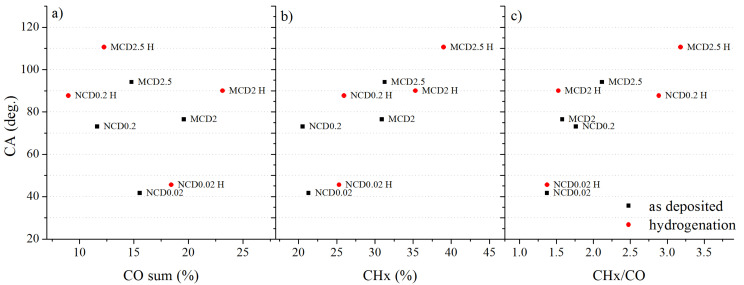
The dependence of CA on chemical composition, before (black squares), and after hydrogenation (red circles). CA versus (**a**) the sum of peaks from oxidised carbon, (**b**) content of CHx bonds and (**c**) ratio of CHx bond content to the sum of oxidized carbon species.

Surface concentration of sp^2^ carbon phase is rather low for all samples—below 10%, possibly in graphite-like structure located in grain boundaries and thus not easily reachable by H radicals. More sp^2^ phase was detected for the sample with higher contribution from oxidized carbons in C1s spectrum. For MCD2 and NCD0.02 samples, in contrast to MCD2.5 and NCD0.2 ones, also a slight increase (a few percentages) in concentration of C–O–C or –C–OH groups was observed after hydrogenation. This increase is probably due to partial adsorption of carbonyl or carboxyl groups resulting from exposition to air during transfer of diamond layers from the CVD chamber to the XPS one. Hydrogen treatment can also restore or improve hydrophobic character of diamond surface exposed to air. Here, for all samples, an increase in contact angle CA was observed, which implies the increase in the number of hydrogen atoms on the surface. For sample MCD2 and MCD2.5 a highly hydrophobic surface character was achieved, CA of 110 deg. (for MCD2.5), whereas for NCD0.2 and NCD0.02, just a reduction of wettability occurred. A decrease in surface wettability can be caused by hydrogen termination, or by an increase in surface roughness. After hydrogenation, indeed, we do observe changes in the surface structure that likely lead to higher roughness; however, it does not exclude the impact from hydrogen termination. Figure 12 shows contact angle dependence on chemical compounds concentration on diamond surfaces.

As one can see, the value of contact angle for MCD2.5 and NCD0.2 varies inversely with the sum of oxygen related peaks (C–O and C=O). Black points indicate data collected before hydrogenation and—red, after this process. For MCD2 and NCD0.02, a reverse trend is observed, which may imply that for these samples, roughness of the surface has an overall greater impact on the final CA value. Figure 12b shows that the contact angle also varies linearly with the CHx concentration; when considering the ratio of CHx bonds to the sum of carbon-oxygen bonds (Figure 12c), the relation becomes even more evident. This CA dependence clearly shows that wettability properties of polycrystalline diamond surface strongly depend on its chemical composition.

## 4. Conclusion

Diamond layers deposited by CVD were studied by Raman spectroscopy, XPS and SEM techniques before and after treatment with hydrogen. As a consequence strong modifications of the surface chemistry and structure were observed for diamond layers. Hydrogen termination of diamond surface was confirmed by XPS and CA measurements, which shows that successful hydrogen termination can be achieved by hydrogen HF process.

In general, the etching by hydrogen atoms leads to a more defective surface, removal of sp^2^ carbon phase and partial conversion of sp^3^ carbon phase into CH_2_ and CH_3_ bonds, as proved by XPS. For diamond layers with higher values of *m/I_G_* parameter also a higher concentration of CHx bonds at the surface was found. At the same time, a decrease in the fraction of t-Pa structures and ordering of amorphous carbon phase in deeper volume of diamond layers were observed. We have shown that post-growth treatment of diamond layers with hydrogen leads to improvement in their photoluminescence properties.

Additionally, we have found that the *m/I_G_* parameter of diamond samples increases with growing hydrogen concentration in the working gas upon deposition. At the same time, the samples with higher H_2_ to CH_4_ ratio exhibit lower contents of trans-Polyacetylene structures when compared to the samples with lower hydrogen concentration. Therefore, *m/I_G_* is likely to be mainly related to the content of C-H bonds in sp^3^ hybridization, and not in sp^2^. Another possible explanation of this observation is different configuration of trans-Polyacetylene in CVD diamond layers with a lower content of C-H bonds.

We have shown that the impact of treatment with hydrogen on diamond layer surface structure depends not only on the size of diamond grans or sp^2^/sp^3^ ratio, but probably also on the preferential orientation of the surface. Moreover, the amorphous carbon layer that was generated during the ion etching process also reacted differently to the treatment with hydrogen, which means that the form of this additional layer had to be different for samples with different grain sizes.

Most likely, a few effects in the hydrogen termination process influence the final results, e.g., high temperature leads to ordering of amorphous carbon phase and reduction of defects; however, the reaction of hydrogen radicals with surface structures causes etching of the amorphous carbon, partial removal of graphitic and t-Pa like structures as well as nanodiamonds from grain boundaries. Therefore, hydrogen treatment leads to strong structure modification of the surfaces of nanocrystalline diamond layers.

In conclusion, diamond layer is more susceptible to changes induced by the hydrogen termination process, when having smaller grain size and higher content of sp^2^ carbon phase. We also would like to emphasize the importance of studying the same area of the sample, when the effects of process such as hydrogenation are considered (but possibly also others like oxidation etc.), as the structural changes can sometimes be very subtle.

## Figures and Tables

**Table 1:** Parameters of diamond layers deposition. The pressure during deposition process was kept at 27 mbar, the filament temperature was kept at 2000 K, and the substrate temperature at 900 K.

Sample	Time of Deposition	CH_4_ Concentration in Working Gas	Average Size of Diamond Grains	Layer Thickness
	h	±0.1%	µm	µm
MCD2	6.0	1.0%	2.0 ± 0.8	3 ± 0.5
MCD2.5	2.5	2.4%	2.5 ± 0.6	3 ± 0.5
NCD0.2	1.0	3.4%	0.2 ± 0.1	2 ± 0.5
NCD0.02	1.0	3.8%	0.06 ± 0.02	2 ± 0.5

**Table 2:** The relative contribution of C1s components before (for as-deposited samples.) and after (H) treatment with hydrogen.

	MCD2		MCD2.5		NCD0.2		NCD0.02	
	As-Deposited	H	As-Deposited	H	As-Deposited	H	As-Deposited	H
sp^2^	10%	7%	3%	3%	4%	4%	3%	6%
sp^3^	39%	34%	51%	46%	64%	61%	60%	50%
CHx	31%	35%	31%	39%	21%	26%	21%	25%
C–O	15%	17%	9%	10%	7%	7%	9%	9%
C=O	5%	6%	4%	2%	3%	2%	4%	5%
C–O–O	0%	0%	3%	0%	1%	0%	3%	4%

## Data Availability

Restrictions apply to the availability of these data. Data are available from the corresponding author with the permission of Poznan University of Technology.
